# Imaging to intervention: a review of what the Interventionalist needs to Know about Hereditary Hemorrhagic Telangiectasia

**DOI:** 10.1186/s42155-021-00270-2

**Published:** 2021-12-09

**Authors:** Stephanie Sobrepera, Eric Monroe, Joseph J. Gemmete, Danial Hallam, Jason W. Pinchot, Claire Kaufman

**Affiliations:** 1 Department of Radiology, University of Washington, 1959 Northeast Pacific Street, Seattle, WA 98195 USA; 2 Department of Radiology, University of Wisconsin, 1675 Highland Avenue, Madison, WI 53792 USA; 3 Department of Radiology, University of Michigan, 1500 E Medical Center Dr, Ann Arbor, MI 48109 USA; 4 Department of Radiology & Imaging Sciences, University of Utah, 30 North 1900 East, Salt Lake City, UT 84132-2140 USA

## Abstract

Hereditary hemorrhagic telangiectasia (HHT) is a disorder that affects 1 in 5000–10,000 people worldwide and can result in devastating complications such as cerebral abscess, stroke, massive hemorrhage, and even death. HHT is an autosomal dominant disorder that leads to the formation of abnormal communication between the arteries and veins with a resultant spectrum of vascular anomalies. The disorder affects many organ systems and thus requires a dedicated multidisciplinary approach. Interventional radiologists are vital members of this team providing expertise not only in disease management, but in complex embolotherapy, helping to maintain the health of these patients. This article reviews clinical manifestations, screening guidelines, diagnostic criteria, and endovascular management of HHT.

## Background

Hereditary hemorrhagic telangiectasia (HHT), also referred to as Osler-Weber-Rendu syndrome, is an autosomal dominant disease that causes abnormal communication of arteries and veins throughout the body (Faughnan et al. 2020; Kim et al. 2015). These abnormal communications lead to a spectrum of manifestations ranging from small cutaneous telangiectasias to large arteriovenous malformations (AVMs). Telangiectasias present clinically as punctate red lesions that are between 1 to 3 mm and are blanchable (McDonald et al. 2011). These appear similar to cherry angiomas,however the key difference is telangiectasias will blanch with pressure. These lesions represent a direct communication between the arteriole and venule without an intervening capillary bed. Telangiectasias can be symptomatic or asymptomatic. They are usually seen involving the oral cavity, nasal mucosa, lips, fingers, and gastrointestinal tract (McDonald et al. 2011; Olitsky 2010). Telangiectasias of the gastrointestinal tract and nasal mucosa tend to be more symptomatic leading to chronic enteric blood loss and epistaxis, respectively (Flieger et al. 2006; Willems et al. 2009; Yen and Chen 2016; Gossage and Kanj 1998).

**Fig. 1:**
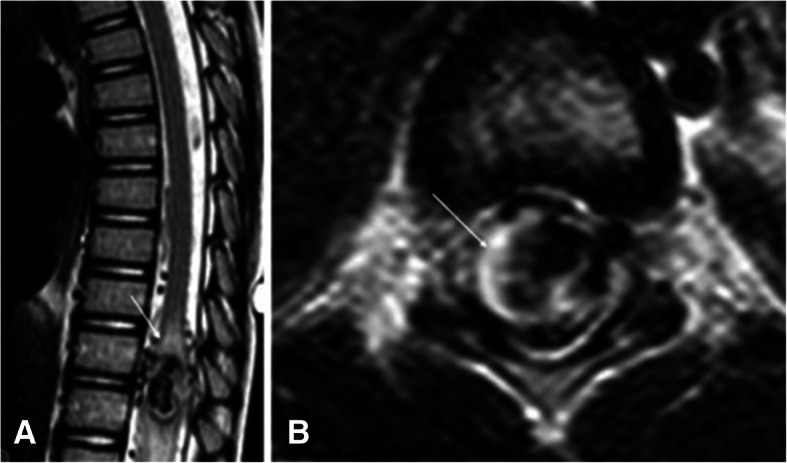
**A** and **B** 3 yo male with left perimedullary fistula. Lateral and axial T2 weighted images of the spinal show similar findings. There is abnormal high T2 signal within the spinal cord (white arrow) surrounding the venous varix

**Fig. 2:**
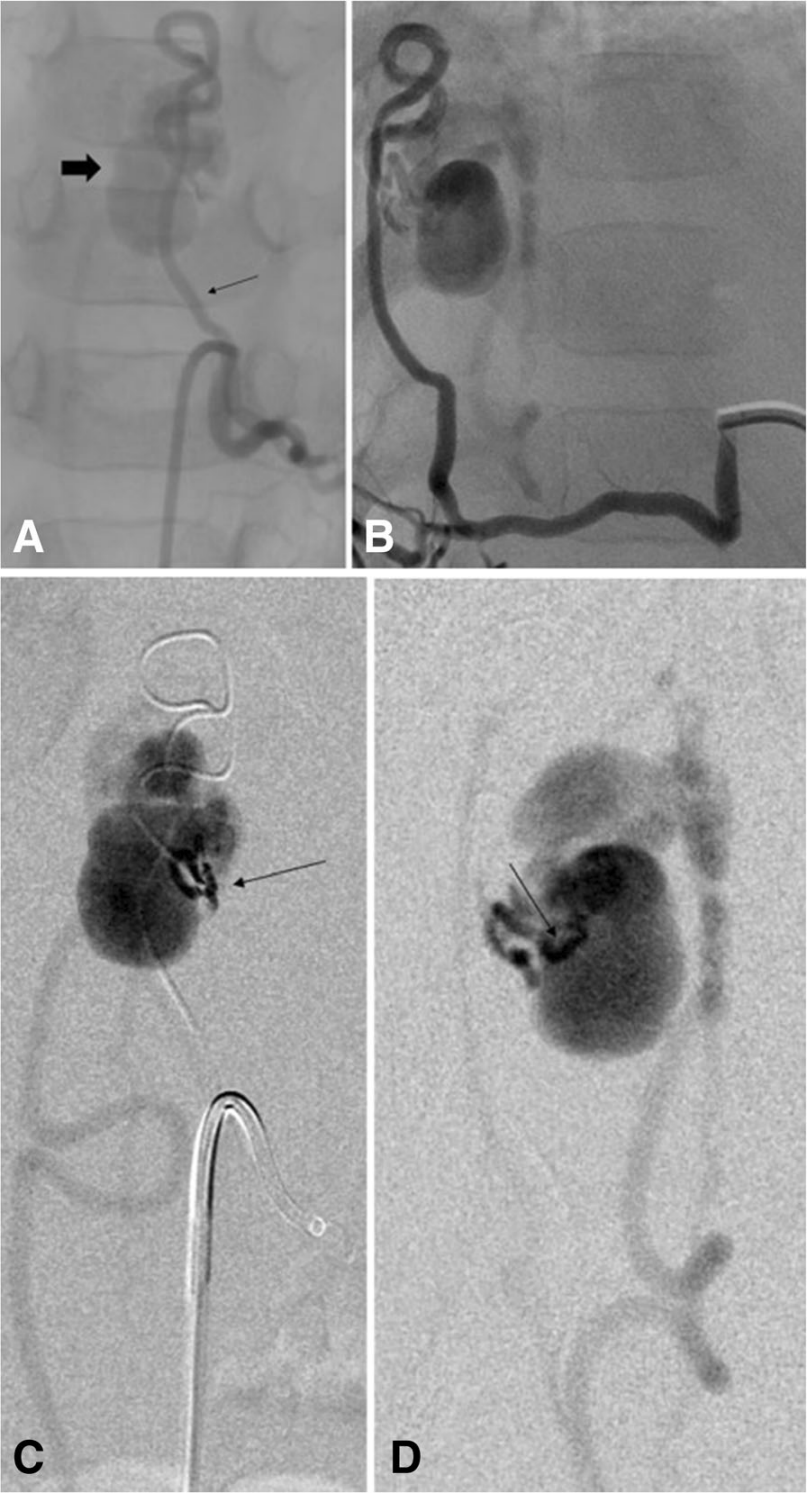
**A** and **B** 3 yo male with left perimedullary fistula. Left T10 intercostal angiogram in the AP and lateral projections shows an enlarged radiculomedullary artery (small arrow) filling a venous varix (thick black arrow) corresponding to the CTA and MRI images. **C** and **D** Selective microcatheter injection in the AP and lateral projections (black arrow) in the venous varix shows filling of the varix and surrounding perimedullary venous plexus

AVMs are higher flow and can have complications related to blood shunting. These most commonly occur in the lungs, liver, and central nervous system (Pollak et al. 2006). In the lungs, these pulmonary AVMs (PAVMs) cause right-to-left shunting. This can lead to hypoxia from poor oxygenation, paradoxical emboli causing abscesses, TIA or strokes, or rupture leading to hemorrhage (McDonald et al. 2011; Pollak et al. 2006; Krings et al. 2015). Cerebral AVMs can cause headache, seizures, and catastrophic intracranial hemorrhage (Meybodi et al. 2018; McDonald et al. 2020; Pawlikowska et al. 2018). Spinal AVMs are less common than cerebral AVMs and often present with back pain or paralysis usually in the first decade of life (Figs. 1, 2). Liver AVMs cause complications from shunting leading to high output cardiac failure, biliary disease, portal hypertension, and less commonly encephalopathy.

This multisystem disease has variable penetrance and expressivity so many patients go undiagnosed for years unless already identified by other family members (Olitsky 2010; Faughnan et al. 2011; Shovlin et al. 2000). Given the potentially severe consequences of failure to recognizing this disease early on, it is crucial to appropriately screen patients with suspicious clinical manifestations and intervene prophylactically as needed.

### Clinical diagnosis

The Curaçao criteria was established in 1999, setting the standard for the clinical criteria for diagnosis of HHT (Table 1) (Shovlin et al. 2000). The four diagnostic criteria include epistaxis, telangiectasias, visceral lesions, and family history of a first-degree relative with HHT. The diagnosis is definite with three criteria met, possible or suspected with two criteria met, and unlikely if fewer than two criteria are present.

**Table 1:** Curaçao Criteria

Criteria	Characteristics
Epistaxis	Spontaneous, recurrent nosebleeds
Telangiectasia	Multiple sites including lips, oral cavity, nose, fingers
Visceral Lesions	Gastrointestinal telangiectasias, pulmonary arteriovenous malformations, hepatic shunting, cerebral and spinal arteriovenous malformations
Family history	First degree relative with HHT

Definite HHT if 3 criteria are present

Possible if 2 criteria are present

Unlikely if < 2 criteria are present

In September 2020, the Second International Hereditary Hemorrhagic Telangiectasia Guidelines were published as an update to evidence-based consensus guidelines (Faughnan et al. 2020). These guidelines are extensive in their recommendations and provide a framework for clinicians to manage specific symptoms. The expert panel continues to recommend the use of the Curaçao Criteria to diagnose HHT which remains widely used today.

### Genetic diagnosis

Several gene mutations have been associated with HHT allowing for focused genetic testing. The majority (50–85%) of cases involve mutations on two genes; Type 1 involves the ENG gene on chromosome 9 (encoding endoglin), while Type 2 involves the ACVRL1/ALK1 gene on chromosome 9 (encoding activin A receptor-like kinase) (Kim et al. 2015; McDonald et al. 2020; Pawlikowska et al. 2018; Brinjikji et al. 2017a; Govani and Shovlin 2009). In addition, a mutation of the SMAD4 gene on chromosome 18 (tumor suppressor gene that mediates TGF-beta) is associated with a combination syndrome involving both juvenile polyposis and hereditary hemorrhagic telangiectasia, seen in approximately 1–2% of tested patients (Faughnan et al. 2011; Brinjikji et al. 2017a; Trerotola and Pyeritz 2010). These gene mutations (ENG, ACRL1/ALK1, SMAD4) encode proteins that modulate transforming growth factor (TGF-beta) signaling involved in angiogenesis (Govani and Shovlin 2009). The phenotypical manifestations of these genes are summarized in Table 2 (McDonald et al. 2015; Gonzalez et al. 2018; Lesca et al. 2007; Hetts et al. 2021).

**Table 2:** Genotype to Phenotype

Variant	Mutation	Epistaxis	GIB	Pulmonary AVM	CNS AVM	Hepatic AVM
HHT-1	*ENG*	++	+	++	++	+
HHT-2	*ACVRL1*	+	++	+	+	++
JP/HHT	*SMAD4*	+	++	+	+	+

*GIB* Gastrointestinal bleeding, *CNS* Central nervous system, *HHT* Hereditary hemorrhagic telangiectasia. *ENG* Endoglin, *ACVRL1* Activin A type-II-receptor-like kinase 1, *JPS* Juvenile Polyposis Syndrome factor 2

Once a family member has been diagnosed with HHT, subsequent relatives only need to be tested for that one mutation. Approximately 15% of patients will have negative genetic testing but a positive clinical diagnosis for HHT (Olitsky 2010; Trerotola and Pyeritz 2010; McDonald et al. 2015). Given the variance of phenotype and expressivity, it is accepted that individuals that have a familial association and have only one Curaçao criterion should get genetic testing (Faughnan et al. 2011). Mutations in ENG and ACVRL1/ALK1 genes were identified in approximately 75% of individuals who met criteria for clinical diagnosis of HHT, confirming good concordance of mutations and disease occurrence (Bossler et al. 2006; Prigoda 2006). The overall prevalence of this disease broadly ranges depending on geography. The prevalence of HHT in Europe ranges from 1 to 5000–10,000 people (Krings et al. 2015; Govani and Shovlin 2009; Franchini et al. 2013; Cartin-Ceba et al. 2013). The prevalence is highest in the Afro-Caribbean population of the Netherlands Antilles at 1 in 1331 in the Curaçao and Bonaire regions (Westermann et al. 2003).

Of note, there is significant phenotypic overlap of capillary malformation-arteriovenous malformation 2 (CM-AVM2), caused by pathogenic variants in EPHB4, and HHT. In patients that are phenotypically suspicious for HHT but test negative for a variant in HHT genes (ENG, ACVRL1/ALK1, or SMAD4), consideration of a pathogenic EPHB4 variant is crucial. However, testing negative for a variant in HHT genes is not necessarily pathognomonic for a concomitant EPHB4 variant and CM-AVM diagnosis. (Wooderchak-Donahue et al. 2019).

### Epistaxis

Epistaxis is the most common manifestation of HHT with approximately 90% of those affected exhibit this clinical symptom by the age of 21 years, and 95% during their lifetime (McDonald et al. 2011; Govani and Shovlin 2009). Nose bleeds can range from occasional few drops on a tissue to heavy, gushing bleeds requiring emergent management (Olitsky 2010; Govani and Shovlin 2009; Franchini et al. 2013). Treatment can be conservative including nasal packing during acute bleeds, or preventative with regular application of ointments to the dry mucosa and humidification of air (Willems et al. 2009). Medical treatments include estrogen, progestogens, tranexamic acid, bevacizumab (Avastin), and even thalidomide (Faughnan et al. 2020; Dupuis-Girod et al. 2012). Current research is examining other possible medications.

Catheter mediated embolotherapy and laser cauterization can be considered for more severe cases of epistaxis that may cause large volume blood loss resulting in iron deficiency anemia. Surgical options are septal dermoplasty, arterial ligature, and nasal cavity obliteration (Young’s procedure)(Willems et al. 2009; Richer et al. 2012; Trojanowski et al. 2011; Strach et al. 2011). The Young’s procedure is used for patients with severe, refractory, transfusion dependent epistaxis and has been shown to lead to complete cessation of epistaxis (Richer et al. 2012).

Anterior bleeding usually arises in Little’s area (also known as Kisselbach’s plexus) along the anterior nasal septum. It is where the anastomosis of the anterior ethmoidal artery, posterior ethmoidal artery, sphenopalatine artery, greater palatine artery, and septal branch of the superior labial artery occurs. Posterior bleeds originate from the sphenopalatine arteries of the nose and are more difficult to control with nasal packing and conservative therapies than anterior bleeding (Willems et al. 2009; Strach et al. 2011). The posterior bleeds have historically been managed with surgical ligation of the sphenopalatine artery or with endoscopic ablation (Willems et al. 2009). In 1974, endovascular treatment of epistaxis was first introduced as an alternative, minimally invasive option to surgery with selection embolization of the maxillary artery with gelatin slurry (Willems et al. 2009; Sokoloff et al. 1974). Angiographic embolization has proven to be a clinically safe and effective treatment of severe epistaxis (Layton et al. 2007; Christensen et al. 2005). Christensen et al. conducted a retrospective review of 70 patients treated with angiographic embolization for posterior epistaxis from 1993 to 2002 and demonstrated an average success and complication rate of 88% and 12%, respectively (Christensen et al. 2005). In a separate study, Strach et al. reported a primary success rate of 93.8% (45/48) for achieving hemostasis by endovascular treatment, comparing to reported failures rates of 26–52% for nasal packing and 4.3–33% for surgical ligation of the internal maxillary artery (Strach et al. 2011; Vitek 1991; Elahi et al. 1995; Tan and Calhoun 1999; Cullen and Tami 1998; Strutz and Schumacher 1990; Metson and Lane 1988; Wang and Vogel 1981; Schaitkin et al. 1987).

**Fig. 3:**
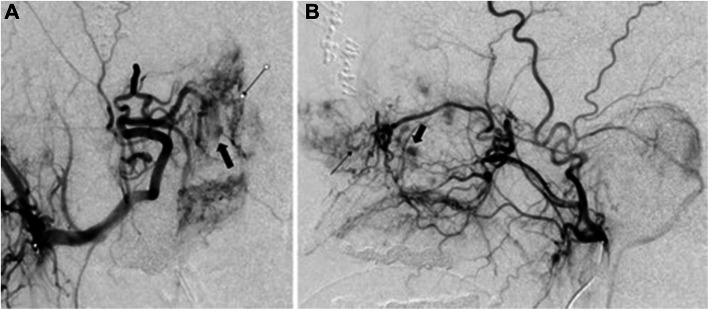
**A** and **B** 45-year-old male who presented with uncontrollable epistaxis. AP and lateral right internal maxillary angiogram show a prominent blush over the right nasal cavity (small black arrow) with areas of prominent pooling of contrast (thick black arrow)

**Fig. 4:**
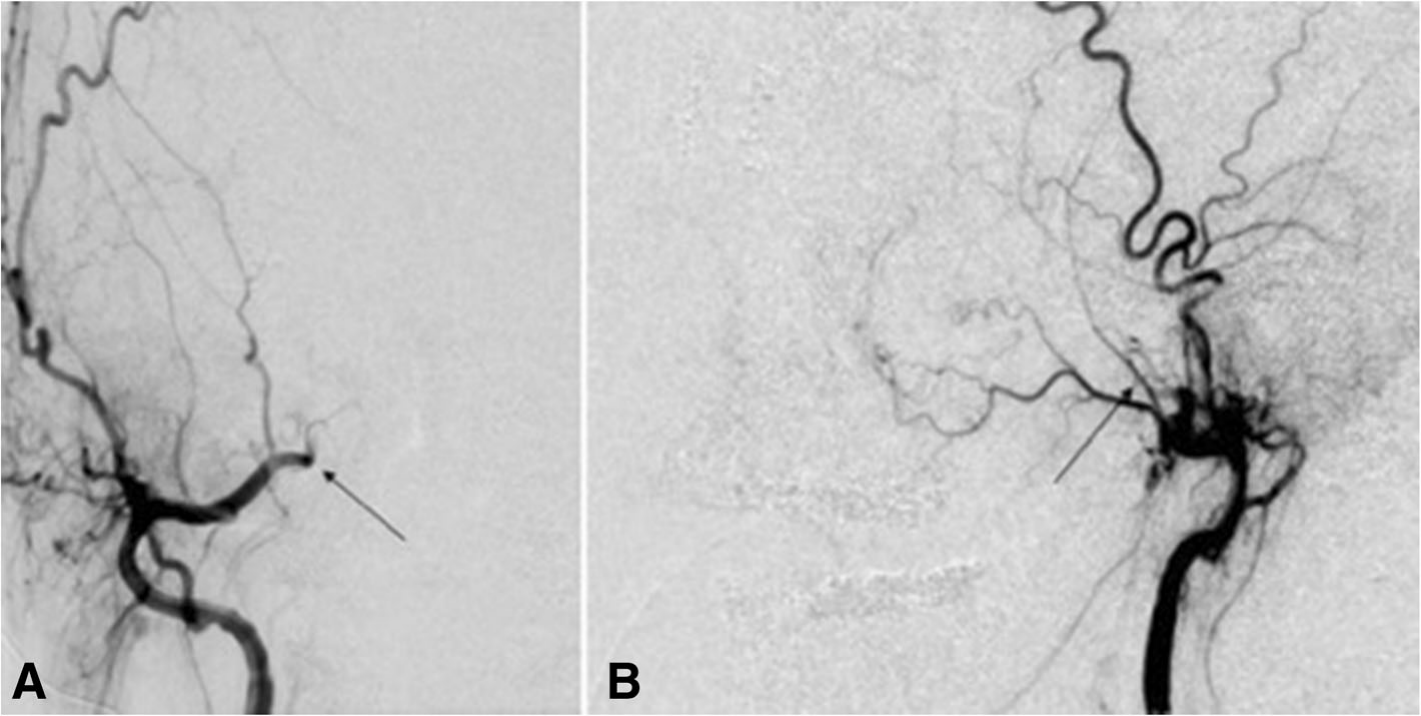
**A** and **B** AP and lateral right internal maxillary angiogram after embolization of the distal branches of the internal maxillary artery with 300–500 μm embospheres and gelfoam pledgets shows truncation of the distal internal maxillary artery (small black arrow) with no vascular blush

A preprocedural diagnostic angiogram of the internal carotid artery (ICA) and external carotid artery (ECA) should be obtained to identify variant anatomy, the location of the bleeding, presence of pseudoaneurysms, or other cause of abnormal bleeding (Willems et al. 2009) (Fig. 3). It is crucial to identify visible connections between the ICA and ECA to decrease risk of nontarget embolization. Even in the cases where there is no visible anastomosis, the interventionalist should note small arteries that may communicate with the ICA with increased pressure during embolization such as the middle meningeal artery, accessory meningeal artery, and anterior pharyngeal arteries (Willems et al. 2009). The goal of embolization is to temporarily decrease flow to the bleeding mucosa without causing tissue necrosis. Caution must be taken to avoid embolization of the ICA, ophthalmic artery, and ascending pharyngeal artery. Placement of the microcatheter in the internal maxillary artery distal to the deep temporal arteries can help in decreasing risk of postembolization pain and trismus. For embolization material, the literature has suggested using varied agents such as polyvinyl alcohol (PVA), gelatin slurry, and calibrated microspheres (300–700 μm) (Willems et al. 2009; Trojanowski et al. 2011; Strach et al. 2011; Sokoloff et al. 1974) (Fig. 4). Endovascular embolization for epistaxis does not provide a long-term cure in most cases due to rebleeding occurring from newly formed telangiectasias and reperfusion. However, the procedure may be repeated if necessary, to provide patients with improved quality of life, reducing the number of hemorrhagic bleeds, or be used as a temporizing procedure prior to more definitive therapy (Layton et al. 2007).

The main contraindication to percutaneous embolization includes unacceptable nontarget embolization. Individuals with ECA-ICA anastomoses in the designated artery for embolization (at risk for cerebral infarction) and those that have bleeding from ethmoidal arteries arising from the ophthalmic artery have increased risk of postinterventional blindness. In these cases, safe embolization is not possible (Strach et al. 2011). The relative contraindications for embolization should also be considered such as severe contrast allergy or coagulopathy.

The most common complications are generally mild including postprocedural facial pain or groin access complications (hematoma, or pseudoaneurysm)More severe complications include facial paresthesia, rebleeding, mucosal necrosis, sinusitis, or non-target embolization (Prigoda 2006; Trojanowski et al. 2011; Layton et al. 2007; Christensen et al. 2005; Oguni et al. 2000). Rarely, devastating complications including blindness, and cerebral infarction can occur (Ashwin et al. 2007). Most of these complications are caused by aggressive embolization leading to reflux of particles back into the ICA and other branches. Gentle and slow injection with magnified continuous fluoroscopy can assist in prevention of reflux of the embolic agent (Strach et al. 2011).

### Pulmonary Arteriovenous malformations

PAVMs are a common finding affecting 80–95% of patients with HHT, preferentially those with Type 1 HHT although they can be seen in all genetic mutations (Cartin-Ceba et al. 2013). PAVMs are high flow and low resistance communications between the pulmonary artery and pulmonary vein, forming an intrapulmonary right-to-left shunt (Cartin-Ceba et al. 2013). The normal capillary bed intervening the artery and vein usually acts as a sieve that filters blood and prevents paradoxical systemic embolization that can cause transient ischemia attacks, strokes, and brain abscesses (Pollak et al. 2006; Faughnan et al. 2000; Mager et al. 2004; Saluja et al. 1999). Rupture of PAVMs is a less common complication but can result in life-threatening hemoptysis and hemothorax (Gossage and Kanj 1998; Swanson et al. 1999).

**Fig. 5:**
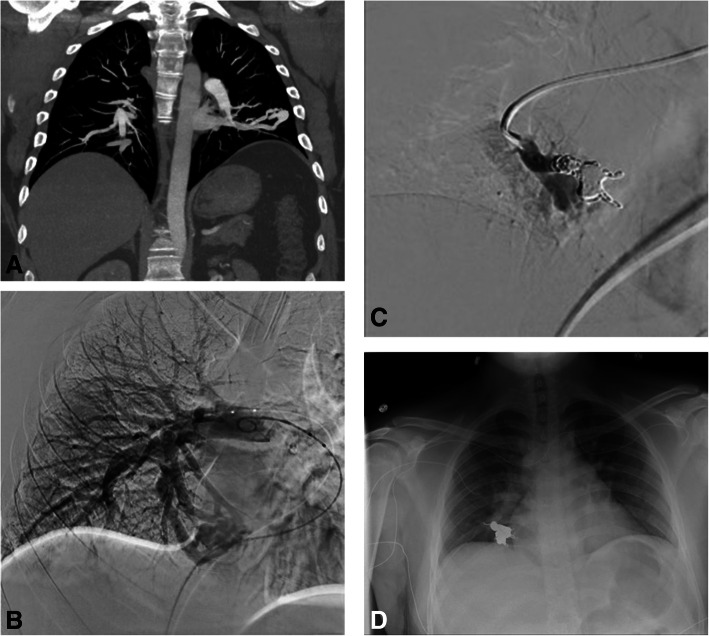
**A** Coronal CT with contrast shown in a maximum intensity projection demonstrates a simple pulmonary AVM in the left lower lobe (arrow). **B** Digital subtraction angiography demonstrating a complex pulmonary AVM in the right middle lobe with multiple feeding subsegmental pulmonary arteries. **C** Digital subtraction angiography post coil embolization of the complex right middle lobe pulmonary AVM demonstrating no flow through the PAVM. **D** Follow-up chest x-ray in the same patient demonstrating the coil pack in the embolized right middle lobe PAVM

PAVMs can be categorized as simple or complex (Fig. 5). Simple PAVMs receive blood through a single feeding segmental pulmonary artery, although there may be multiple subsegmental branches supplying the PAVM. This is in comparison to complex PAVMs which have multiple feeding pulmonary arteries from more than one pulmonary segment (Pollak et al. 2006; Swanson et al. 1999). Generally, the afferent supply is from a pulmonary artery branch, although it is possible to have systemic arterial supply from the bronchial or intercostal arteries especially in cases of reperfusion of a previously embolized PAVM (Cartin-Ceba et al. 2013; Kaufman et al. 2017). The efferent flow is almost always a branch of the pulmonary vein, although communication with the inferior vena cava and left atrium has been rarely reported (Cartin-Ceba et al. 2013).

Initial screening should include a thorough physical exam with oxygen assessment followed by a transthoracic contrast echocardiogram (echo bubble) with agitated saline (Trerotola and Pyeritz 2010; Swanson et al. 1999; Shovlin 2014; White 1992). If there is a positive echo bubble signifying an intrapulmonary shunt, then a CT chest should be obtained to evaluate for presence of size and location of PAVMs (Trerotola and Pyeritz 2010; Shovlin 2014). If treatable PAVMs are identified, pulmonary angiography and intervention should follow (White et al. 1996; Trerotola et al. 2009). Follow-up imaging protocols post-treatment vary slightly by practice, however generally a CT Chest with intravenous contrast should be performed in the first 6–12 months after transcatheter embolization and then every 3–5 years after (Shovlin 2014). PAVMs that are too small to treat require surveillance, which is often done with a chest CT every 3–5 years (Cartin-Ceba et al. 2013).

The treatment of choice for PAVMs is transcatheter embolization (Mager et al. 2004; Shovlin 2014; Lee et al. 1997). Before this minimally invasive approach, the only available therapies until 1977 had been surgical resection or ligation which have significant morbidity associated and the clear disadvantage of losing normal lung parenchyma around the PAVM (Mager et al. 2004; Lee et al. 1997; Haitjema et al. 1995; White et al. 1988). Transcatheter embolization is generally safe with few complications (Gossage and Kanj 1998; Haitjema et al. 1995). These complications include non-target embolization of embolic agent, clot, or air through the PAVM sac and into the systemic circulation leading to stroke. Pleuritic chest pain is often seen after embolization, and is usually self-limited with no intervention required (Mager et al. 2004; Haitjema et al. 1995; White et al. 1988). Up to 25% of technically successful embolizations will require re-treatment due to reperfusion (Majumdar and McWilliams 2020). Reperfusion can occur through recanalization of previously placed coils, via recruitment of adjacent pulmonary arteries, or via recruitment of systemic collaterals (Kaufman et al. 2017). The goal of embolization is to reduce the right-to-left shunt, thereby improving arterial oxygenation and decreasing or eliminating the risk for paroxysmal embolization and subsequent complications, as well as rupture (Gupta et al. 2002).

It had been historically believed that PAVMs with feeding arteries of 3 mm or more should be treated due to the risk of paradoxical embolism (Gossage and Kanj 1998). However, more recently it has since been recognized that paradoxical embolization can occur with feeding pulmonary arteries smaller than 3 mm with reports in the literature of neurologic complications (Andersen and Kjeldsen 2012; Todo et al. 2004). With the introduction of modern technology including microcatheter systems and detatchable microcoils and plugs, it is now possible and recommended to embolize smaller pulmonary AVMs below 3 mm in diameter as stated in the 2011 International Guidelines for Diagnosis and Management of HHT (Faughnan et al. 2011; Trerotola and Pyeritz 2010; Cartin-Ceba et al. 2013; Trerotola et al. 2009; Andersen and Kjeldsen 2012).

There are several techniques to embolize an AVM – all of which have the goal to embolize the feeding artery as distal as possible to avoid occlusion of branches that supply normal lung parenchyma and decrease the chances of reperfusion. The choice of embolic depends on the size and configuration of the PAVM, but should be mechanical obstruction with coils or vascular plugs - never particles or liquid embolics (Trerotola et al. 2009; Andersen and Kjeldsen 2012). One study reportsing the use of an Amplatzer vascular plug with at least one platinum coil in addition, showed no recanalization at mean follow up time of 13 months (Trerotola and Pyeritz 2010).

Reperfusion of the treated PAVM can occur from recanalization, interval growth of an accessory vessel, or collateral reperfusion with rates ranging from 5 to 57% (Kaufman et al. 2017; Woodward et al. 2013; Sagara et al. 1998). The treatment of PAVMs, either via transcatheter or surgical approaches, will not prevent the growth of small pulmonary AVMs or stop the formation of new ones (Swanson et al. 1999). Because of this, in addition to the high rate of reperfusion, it is important to ensure patients have appropriate lifelong follow-up to manage their care even after successful embolization(Trerotola et al. 2009).

### Hepatic AVM

**Fig. 6:**
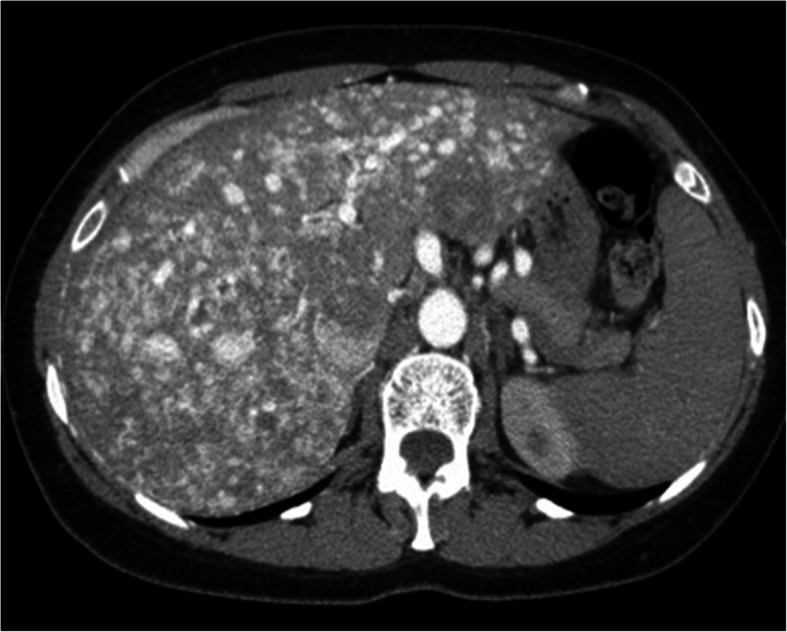
Axial CT with contrast of a 45-year-old woman with HHT demonstrates innumerable enhancing arteriovenous malformations throughout the liver parenchyma. Partially visualized is hypertrophy of the celiac artery

**Fig. 7:**
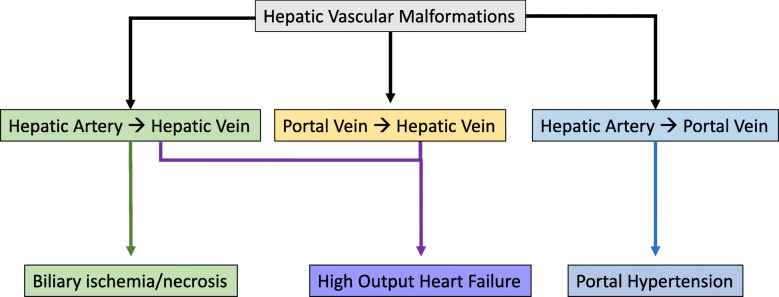
Manifestations of Hepatic Vascular Malformation Shunts. Hepatic artery to hepatic vein shunts can result in biliary ischemia and necrosis due to the single blood supply to the biliary system from the peribiliary plexus via the hepatic artery. Shunting to the hepatic vein such as through hepatic artery to hepatic vein shunting and portal vein to hepatic vein shunting, contribute to high output heart failure. Hepatic artery to portal vein shunts leads to portal hypertension

Liver AVMs are relatively common and have a prevalence between 41 and 78% (Buscarini et al. 2006) (Fig. 6). These lesions are typically clinically silent (Buscarini et al. 1997; Lerut et al. 2006). Complications from liver AVMs occur in approximately 8% of people with HHT and result from shunting of the hepatic artery to hepatic vein, portal vein to hepatic vein, and/or hepatic artery to portal vein (Buscarini et al. 2006) (Fig. 7). High output cardiac failure is the most common complication of intrahepatic shunting with symptoms generally reported when intrahepatic shunt output is greater than 20% of the cardiac output (Buscarini et al. 2006). Symptoms include dyspnea on exertion, ascites, edema, and even a bruit over the liver (Garcia-Tsao et al. 2000). A shunt that leads to high output cardiac failure involving hepatic artery to hepatic vein and can also cause biliary ischemia due to steal(Lerut et al. 2006). While middle-aged women are the most affected demographic, heart failure can be precipitated by pregnancy due to increased hemodynamic demands and increase in circulating blood volume (Shovlin 2014; Bari and Cohen 2017).

With intrahepatic shunting, the biliary system is at risk due to its single blood supply from the hepatic artery via the peribiliary plexus. Arteriovenous shunting can cause ischemia of the bile ducts which leads to strictures, dilation, and biliary cysts (Garcia-Tsao 2007). Clinical presentation of biliary ischemia includes right upper quadrant pain, cholestasis, and elevated alkaline phosphatase and gamma glutamyl transpeptidase (Buscarini et al. 2006; Garcia-Tsao 2007). The most severe form of biliary ischemia is hepatic disintegration, characterized by disruption of the structure of the liver with hepatocyte necrosis leading to cholangitis, hemorrhage, and bile leak (Buscarini et al. 2006).

**Fig. 8:**
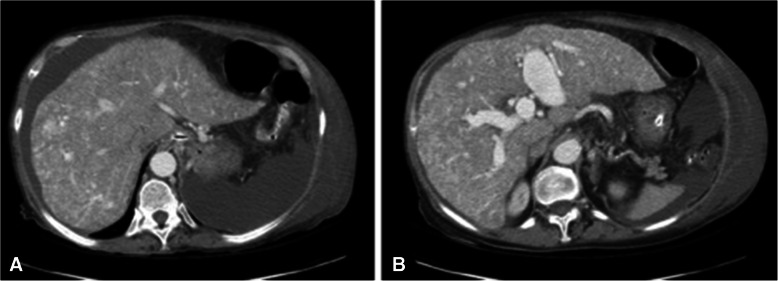
**A** and **B** Axial CT images with contrast of a 77-year-old female with HHT and portal hypertension showing cirrhotic features due to pseudocirrhosis. Findings consistent with HHT and portal hypertension including ascites, hypertrophy of the caudate lobe, portal venous enlargement, and varices

Portal hypertension is another complication of hepatic AVMs (Fig. 8). It is caused by shunting from the hepatic artery to portal vein or from nodular regenerative hyperplasia that is induced by the altered blood flow (Buscarini et al. 2006). Clinical presentation of portal hypertension is not specific to the underlying cause and includes ascites, varices with or without variceal hemorrhage, and encephalopathy. Pseudocirrhosis can occur due to the hepatocellular regeneration nodules and fibrosis surrounding the vessels which can lead to a misdiagnosis of cirrhosis (Buscarini et al. 2006). The difference between cirrhosis and pseudocirrhosis is the lack of elevation in liver function tests in pseudocirrhosis (Garcia-Tsao 2007).

The diagnosis of hepatic AVMs is often incidental and depend on recognizing the secondary clinical signs such as heart failure, biliary disease, or portal hypertension in patients with known HHT, prompting further work-up. Doppler ultrasound should be used as first line imaging (Faughnan et al. 2020; Buscarini et al. 1997).

Medical treatment is only indicated in symptomatic patients and targets specific symptoms. High output heart failure responds to salt-restricted diets, diuretics, antihypertensives, and antiarrhythmics (Garcia-Tsao et al. 2000). Biliary duct ischemia causing cholangitis is treated with antibiotics. There has been preliminary data using bevacizumab (Avastin), an antiangiogenic medication often used for epistaxis, that has been associated with decrease in cardiac output, decreased number of epistaxis episodes, and reduced duration of epistaxis (Flieger et al. 2006; Dupuis-Girod et al. 2012; Garcia-Tsao 2007).

Hepatic AVMs have historically been “don’t touch lesions” from an endovascular or surgical perspective. Portal decompressive interventions such as transjugular intrahepatic portosystemic shunt (TIPS) have little published experience in treating hepatic AVMs with concern that increased shunting would worsen cardiac output, as well as the puncture itself being high risk (Lee et al. 1998). The only endovascular treatment that might be considered is transarterial embolization, which can be used for treating high output cardiac failure and portal hypertension (Buscarini et al. 1997). This procedure should only be considered in patients with severe intractable symptoms due to the high morbidity and mortality. Complication rates range from 20 to 60%, with the most common complications being biliary or hepatic necrosis (Chavan et al. 2013). Therefore, this procedure is contraindicated in patients with biliary disease and should not be considered unless the patient is not a transplant candidate and has failed medical therapy.

Surgical ligation has the same indications and complications as transarterial embolization (Garcia-Tsao et al. 2000). The only curative treatment for hepatic AVMs is orthotopic liver transplant (Garcia-Tsao 2007). The transplant procedure in these patients is particularly difficult due to the increased intraoperative bleeding from AVMs, so a thorough pre-transplant workup is required to minimize risk. Identifying large pulmonary AVMs is crucial to the pre-transplant workup with subsequent treatment of these lesions prior to transplant to prevent risk of hemorrhage (Lerut et al. 2006). The reported post-operative survival rate of liver transplant in HHT is approximately 80% at 58 month follow up (Garcia-Tsao 2007).

### Gastrointestinal bleeding

Bleeding in the gastrointestinal (GI) tract is due to telangiectasias in the bowel wall. It most commonly presents as an upper GI bleed and are often multifocal (Jackson et al. 2017). The onset of GI bleeding usually starts after the age of 30 years old and increases with age (Govani and Shovlin 2009). When evaluating a patient for GI bleeds, it is important to note that the fecal occult blood test is non-specific because it can result in a false positive due to swallowed blood from epistaxis. More fool-proof methods for diagnosing GI bleeding from telangiectasias are endoscopy, capsule enteroscopy, angiography, or nuclear medicine tagged RBC scans (Faughnan et al. 2020; Jackson et al. 2017). Most often, these GI bleeds can be managed conservatively with antifibrinolytics, octreotide to reduce portal pressures by decreasing splanchnic flow, and aggressive iron therapy with target ferritin goal of greater than 50–100 ng/ml (Lee et al. 1998; Houghton et al. 2019; Tortora et al. 2019). Areas of bowel with focal bleeding or larger malformations can be treated by endoscopy or resected surgically (Yen and Chen 2016). There is a paucity of literature to support catheter directed embolization in the routine treatment of GI telangiectasias, likely due to the small, multifocal lesions leading to slow-flow, and intermittent bleeding not amenable to embolization.

### Cerebrovascular AVM

Depending on the population of interest, 2–20% of patients with HHT have Cerebral AVMs (CAVMs) which can occur in multiplicity (Brinjikji et al. 2017a; Bharatha et al. 2012). People with HHT type 1 are more likely to have CAVMs with a prevalence of 13.4% compared to 2.4% in HHT type 2 (Brinjikji et al. 2017a). CAVMs are a common finding in people that do not have the diagnosis of HHT. Only approximately 10% of patients with CAVMs have a diagnosis of HHT (Shovlin and Letarte 1999). However, multiple cerebral AVMs is a stronger predictor for HHT verses other causes of CAVMs (Bharatha et al. 2012).

CAVMs carry a very high risk of rupture and hemorrhage that can result in severe neurologic deficits and even death (Swanson et al. 1999; Fulbright et al. 1998). Neurological features that have been reported include clinical stroke, transient ischemic attack, brain abscess, and migraine (Swanson et al. 1999; White et al. 1988; Kjeldsen et al. 2014). Approximately 10–20% of HHT patients with CAVMs will present with AVM-related hemorrhage (Brinjikji et al. 2017a; Brinjikji et al. 2016). CAVMs that have high shunt flow, venous outflow obstruction, and intranidal aneurysms are at increased risk of rupture and warrant treatment (Fulbright et al. 1998). Most neurological symptoms in HHT occur due to paradoxical emboli from PAVMs rather than CAVMs (Moussouttas et al. 2000; Kjeldsen et al. 2000).

**Fig. 9:**
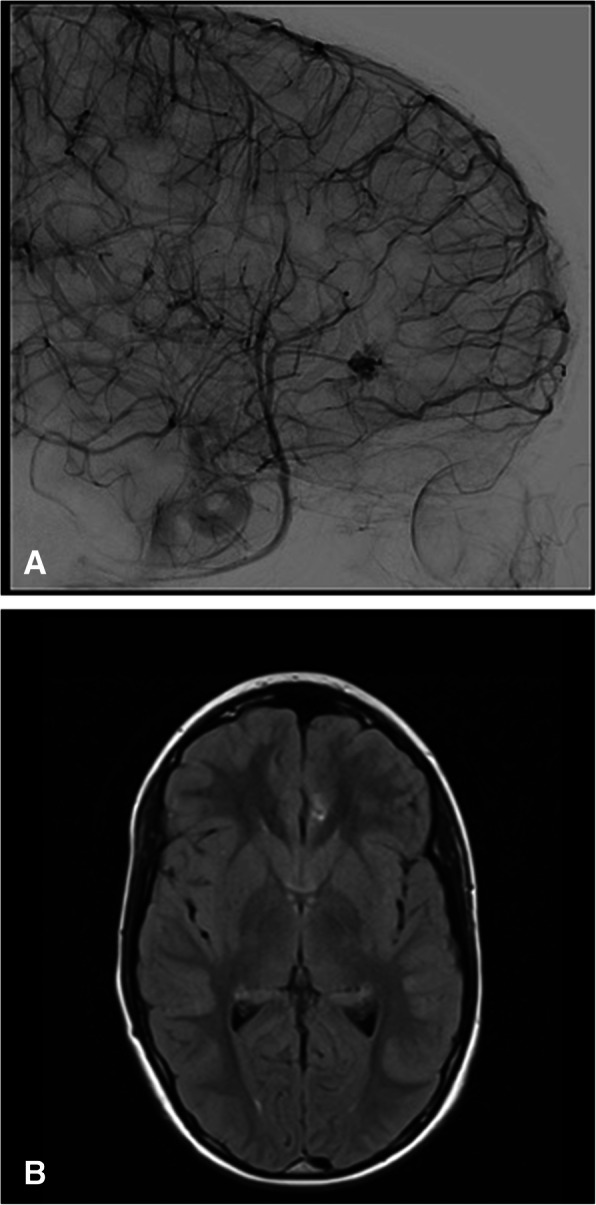
**A** and **B** 12-year-old boy with heterozygous ENG mutation hereditary hemorrhagic telangiectasia and a cerebral capillary vascular malformation. **A** Late arterial phase lateral projection digital subtraction angiogram of the left internal carotid artery demonstrates a sub centimeter focus of ectasia and blush without arteriovenous shunting in the medial aspect of the left frontal lobe (arrow). **B** Axial FLAIR sequence showing corresponding focus (arrow)

**Fig. 10:**
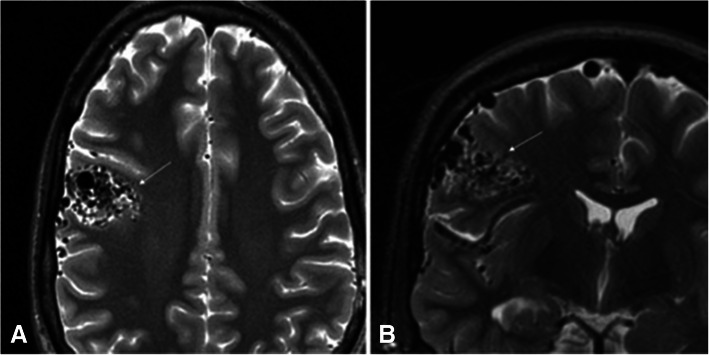
32-year-old male who present with a headache. Axial and Coronal T2 weighted images show a cortical nidal AVM within the posterior right frontal lobe measuring 2.5 cm (white arrow)

There is a spectrum of cerebrovascular lesions associated with HHT which can be categorized into high-flow pial fistulas, nidus-type brain AVMs, and capillary vascular malformations (Brinjikji et al. 2016) (Figs. 9, 10). Nidal brain arteriovenous malformations and capillary vascular malformations occur in nearly equal numbers (Brinjikji et al. 2016). The distinction between the different types of CAVMs is important in predicting the course of disease in the patient. For example, capillary vascular malformations are thought to have a benign course without risk of rupture, whereas nidus type have increased risk of rupture (Brinjikji et al. 2016; Brinjikji et al. 2017b). Additionally, patients that present with intracranial rupture are at higher risk for rebleeding compared to those with CAVMs detected prior to neurological symptoms (Kim et al. 2015).

The protocol for screening, indications, and timing of treatment are not well established, however, due to the relatively high prevalence of CAVMs in HHT patients, a screening MRI study is recommended by most experts in patients with known or suspected HHT (Faughnan et al. 2020; Brinjikji et al. 2017a; Brinjikji et al. 2015). Often children of known families with HHT are screened with MRI in the first year of life (Brinjikji et al. 2015). Once an adult has had a negative screening MRI there is no need to follow (Brinjikji et al. 2015). Treatment can include embolization, stereotactic radiosurgery (Gamma knife), or open surgery. Although of note, there is limited literature on this topic and therefore no accepted standardized model for treatment (Meybodi et al. 2018). There has also been no statistically significant difference in long term outcomes between the surgically and non-surgically treated lesions (Meybodi et al. 2018). According to the updated International HHT guidelines, an individualized management approach for HHT-related cerebrovascular lesions is recommended (Faughnan et al. 2020).

### Management and follow-up

HHT is a complex disease process that has variable symptoms and presentations that require a multidisciplinary team which can include gastroenterologists, hematologists, pulmonologists, cardiologists, interventional radiologists, genetic counselors, and many more. Multiple screening studies are recommended after a diagnosis of HHT (Table 3). According to the HHT International Guidelines, it is recommended that patients with suspected HHT-related organ involvement be treated at a center with HHT expertise because of the multi-faceted nature of this disease (Faughnan et al. 2011; de Gussem et al. 2020). HHT Centers of Excellence are institutions that have an integrated team of experts that are knowledgeable in the diagnosis, treatment, and management of HHT. These centers are certified to provide appropriate screening, treatment, education, and life-long follow up to these patients with the goal to reduce morbidity and mortality (de Gussem et al. 2020).

**Table 3:** Screening Imaging

Anatomy	Initial screening	Follow up
Brain	Children: MRI brain within 1st year of life Adults: MRI with and without contrast with blood sensitive sequence Pregnant women: unenhanced MRI brain	Children with CAVM require serial imaging Adults with negative MRI brain do not require further follow up
Lung	Transthoracic echocardiogram to evaluate shunting Chest CT is not standard in PAVM screening, but can be used to evaluate patients with high suspicion of PAVM CT arteriography no longer necessary as screening, generally reserved for therapeutics	Initial chest CT 6–12 months post treatment, then 3–5 years after If PAVM is not treated, surveillance Chest CT every 3–5 years
Liver	Doppler ultrasound if exhibiting signs and symptoms of complicated liver VMs	

*CAVM* Cerebral arteriovenous malformation, *PAVM* Pulmonary arteriovenous malformation

## Conclusion

HHT is an autosomal dominant disorder characterized by abnormal communications between the arterial and venous systems that affects numerous organ systems. There are many nuances to the disease requiring a multi-disciplinary approach. Interventional radiologists are key members of this team and are often at the forefront of management and treatment of these patients.

## Data Availability

Not applicable.
